# Supplementary figures and images for: Short-range human cortico-cortical white matter fibers have thinner axons and are less myelinated compared to long-range fibers despite a similar g-ratio
Source: PLoS Biol. 2025 Aug 20;23(8):e3002906. doi: 10.1371/journal.pbio.3002906 (PMC12410883; doi:10.1371/journal.pbio.3002906)

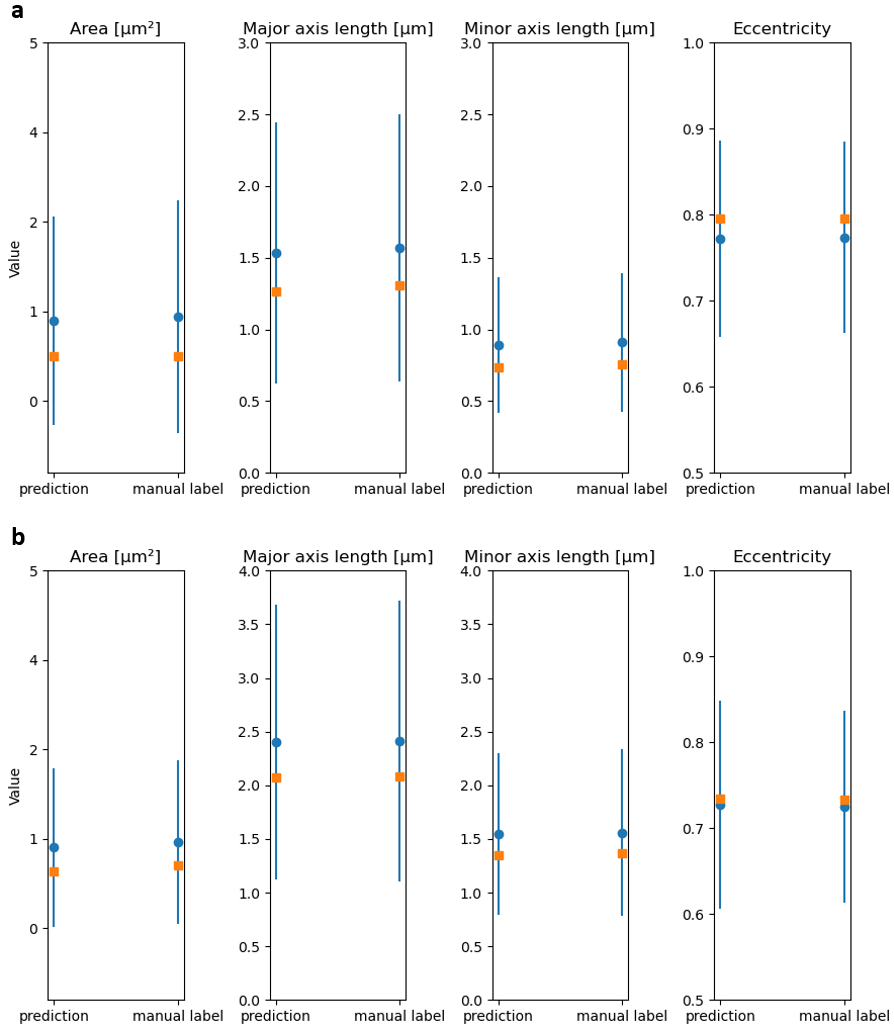

Supplement: S1 Fig — This plot shows population-level differences in the training data (manual label) and our prediction on the same image. The image used was not used for the training of the validation model. The comparison shows only minor systematic biases. All plots show mean ± sd (blue) and median (orange) of area, major and minor axis length and eccentricity of CC axons (a), CC outer fiber measures (b). Data available in S5 Data (https://zenodo.org/records/15720452, [72]). (PNG) [file pbio.3002906.s001.png]

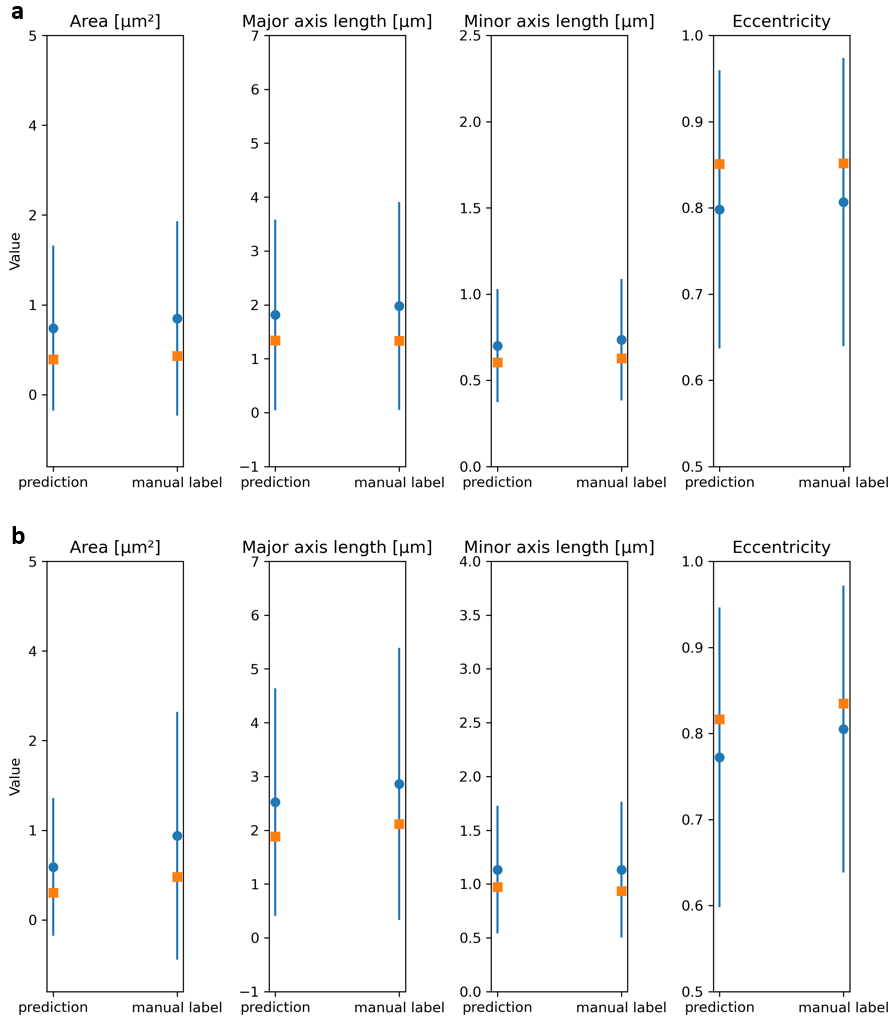

Supplement: S2 Fig — This plot shows population-level differences in the training data (manual label) and our prediction on the same image. The image used was not used for the training of the validation model. The comparison shows only minor systematic biases. All plots show mean ± sd (blue) and median (orange) of area, major, and minor axis length and eccentricity of SWM axons (a), SWM outer fiber measures (b). Data available in S4 Data (https://zenodo.org/records/15720452, [72]). (PNG) [file pbio.3002906.s002.png]

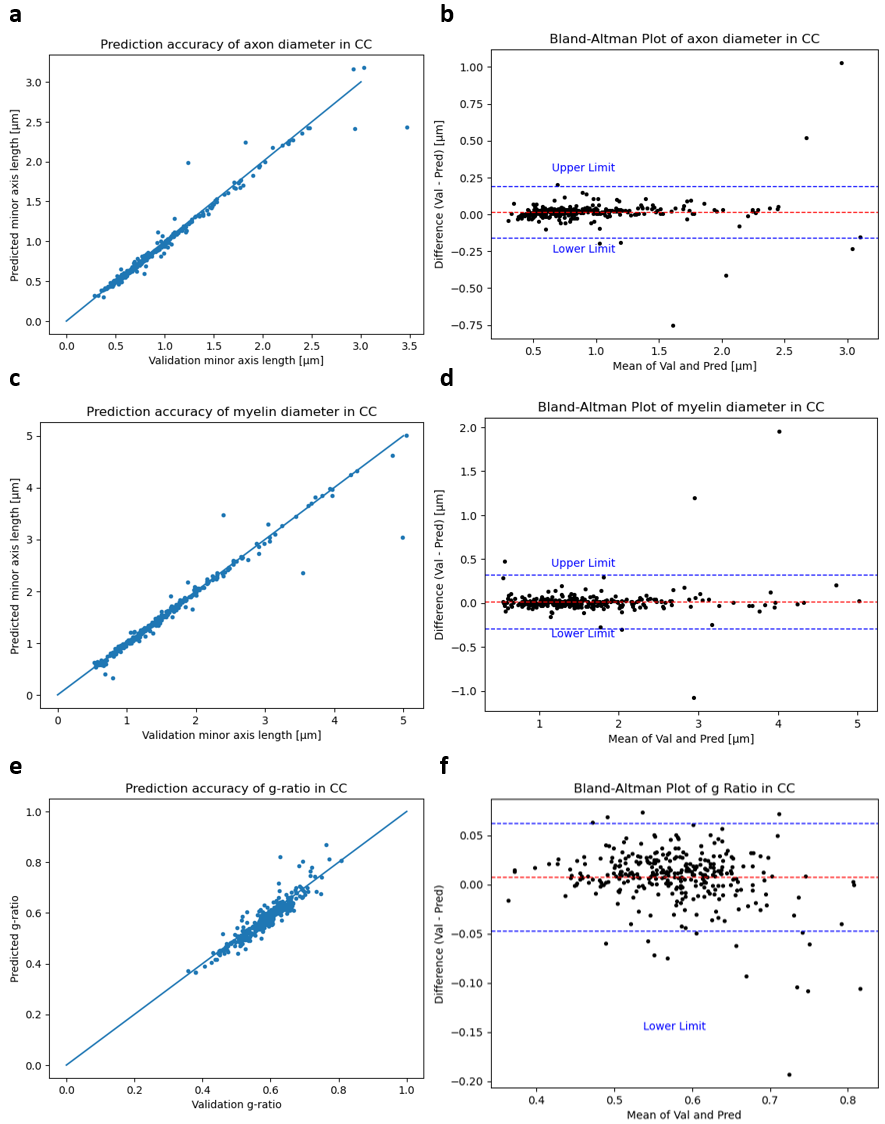

Supplement: S3 Fig — In addition to population-level measures (Figs 1 and 2), we quantified individual biases by matching each structure in the predicted data to the corresponding structure in the validation data (more than 40% overlap with the corresponding validation structure). Plotted blue diagonal (left) and dashed red (right) lines show theoretical perfect segmentation without any bias. Plots show the correlation of predicted measures of each structure with the validation data (left column) and Bland-Altman plots (right column) of CC axon diameter (a,b), CC outer fiber diameter (c,d), CC g-ratio (e,f). The Bland-altman plots show that most points are within limits of agreement, indicating good agreement between manually labeled data and the automated prediction. Data available in S5 Data (https://zenodo.org/records/15720452, [72]). (PNG) [file pbio.3002906.s003.png]

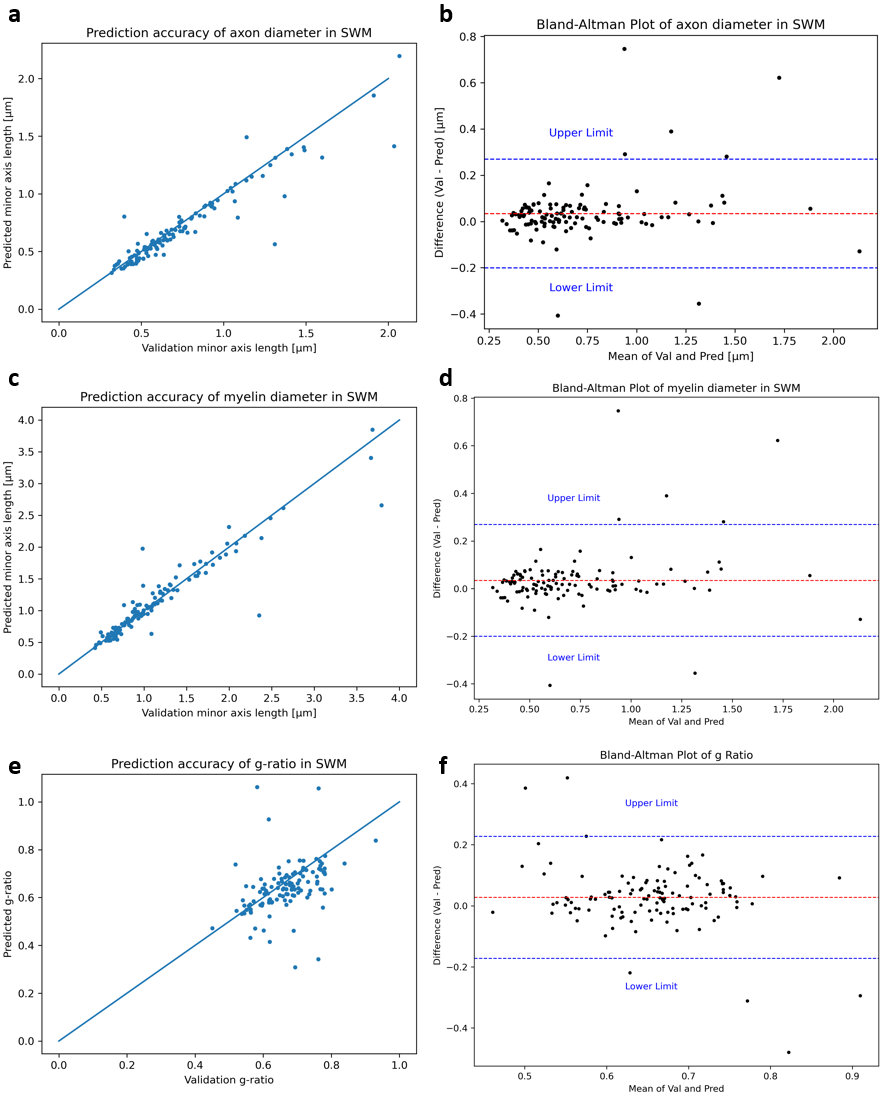

Supplement: S4 Fig — In addition to population-level measures (Figs 1 and 2)), we quantified individual biases by matching each structure in the predicted data to the corresponding structure in the validation data (more than 40% overlap with the corresponding validation structure). Plotted blue diagonal (left) and dashed red (right) lines show theoretical perfect segmentation without any bias. Plots show the correlation of predicted measures of each structure with the validation data (left column) and Bland-Altman plots (right column) of SWM axon diameter (a,b), SWM outer fiber diameter (c,d), SWM g-ratio (e,f). The Bland-altman plots show that most points are within limits of agreement, indicating good agreement between manually labeled data and the automated prediction. Data available in S4 Data (https://zenodo.org/records/15720452, [72]). (PNG) [file pbio.3002906.s004.png]

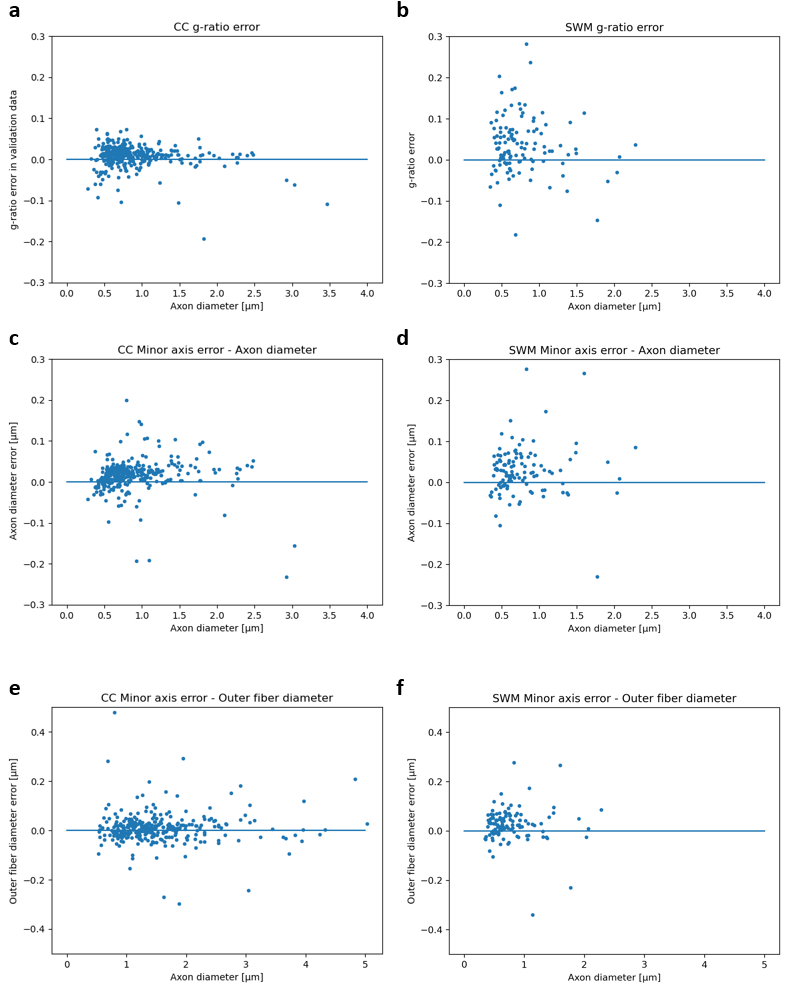

Supplement: S5 Fig — These plots show the axon diameter of the fiber plotted against the measurement error (delta of validation − prediction value) of that structure to show potential biases in particular groups of small or large fibers. These paired error values are shown for g-ratio in CC (a) and SWM (b), axon diameter in CC (c) and SWM (d), and outer fiber diameter in CC (e) and SWM (f). In all cases, the quantified error is far lower than the quantified difference between CC and SWM. Data available in S4 and S5 Data (https://zenodo.org/records/15720452, [72]). (PNG) [file pbio.3002906.s005.png]

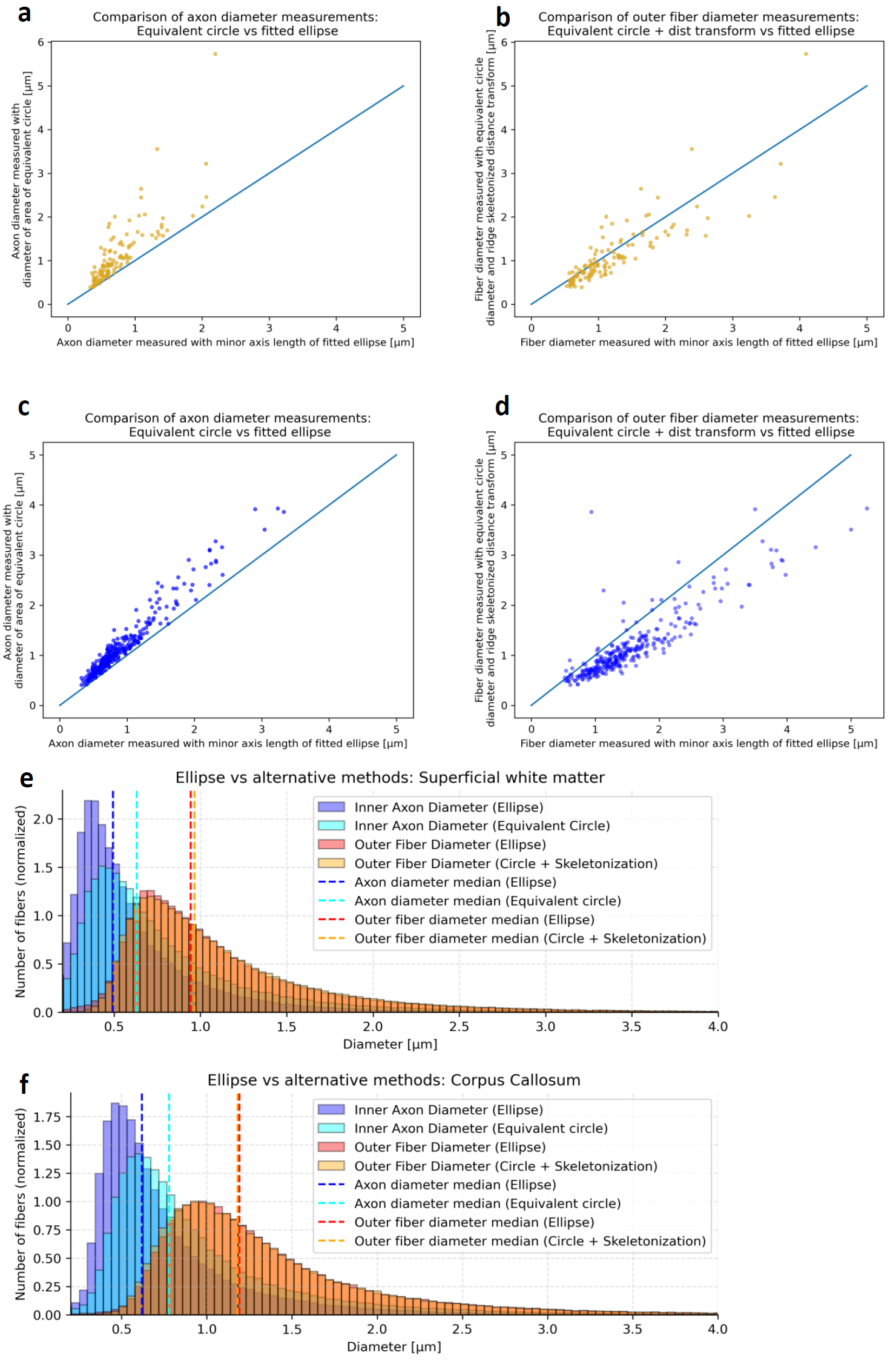

Supplement: S6 Fig — Scatter plots show individually paired results of two different methods for determination of axon diameter (a,c) and outer fiber diameter (b,d) in SWM (a,b) and CC (c,d). One method (plotted on the x axes) generated the results by fitting ellipses to the segmented structures and measuring the minor axis of the fitted ellipse, and the other method (plotted on the y axes) utilized a method analogous to the morphometry analysis conducted in Behanova and colleagues [56]. In brief, the axon diameter was measured by calculating the diameter of the equivalent area circle, and the myelin thickness was measured by distance transformation of the masked myelin. Both were added together for b and d. As expected, measuring the area of the equivalent circle for axon diameter increases the measured areas, due to oblique cuts not being corrected for (CC: +27%, SWM: +48%). Examining the difference between these methods on a data that is not instance-matched (e,f), we also find that axon diameter is overestimated when measuring the radius of the area-equivalent circle compared to measurements taken on the minor axis of the fitted ellipse. In contrast, the outer fiber diameter of measuring the fitted ellipse is almost identical to the outer fiber diameter estimated using the equivalent circle radius and skeletonizing myelin analogous to Behanova and colleagues [56]. Data available in S4 and S5 Data (https://zenodo.org/records/15720452, [72]). (PNG) [file pbio.3002906.s006.png]

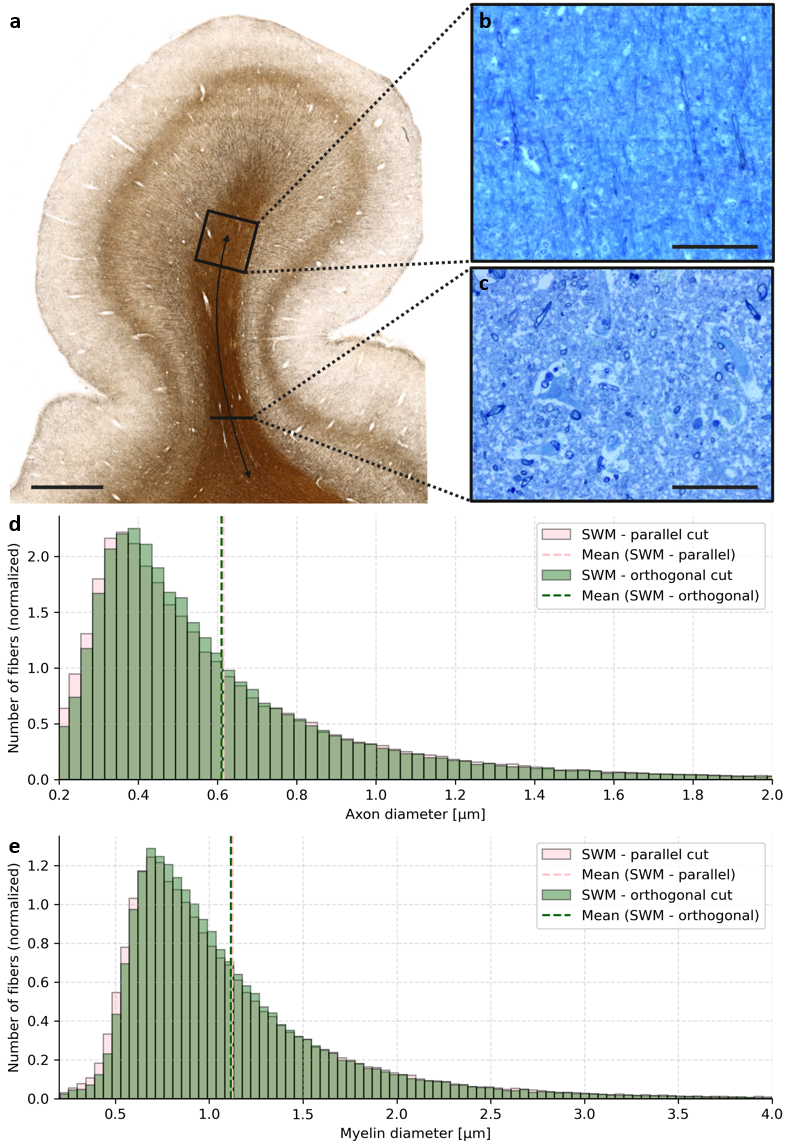

Supplement: S7 Fig — a) shows an exemplary fiber stained slice of human SWM (gallyas silver staining), with cutouts showing (b) parallel and (c) orthogonally cut sections of SWM. The arrow indicates the main fiber direction. (b,c) show toluidine blue stained sections, with b showing more longitudinally/diagonally cut fibers than c. (d,e) Axon diameters (d) and outer fiber diameters (e) of SWM data, split according to cutting angle. Measurements were taken from TEM data, as described in Methods. Data available in S3 Data (https://zenodo.org/records/15720452, [72]). (PNG) [file pbio.3002906.s007.png]

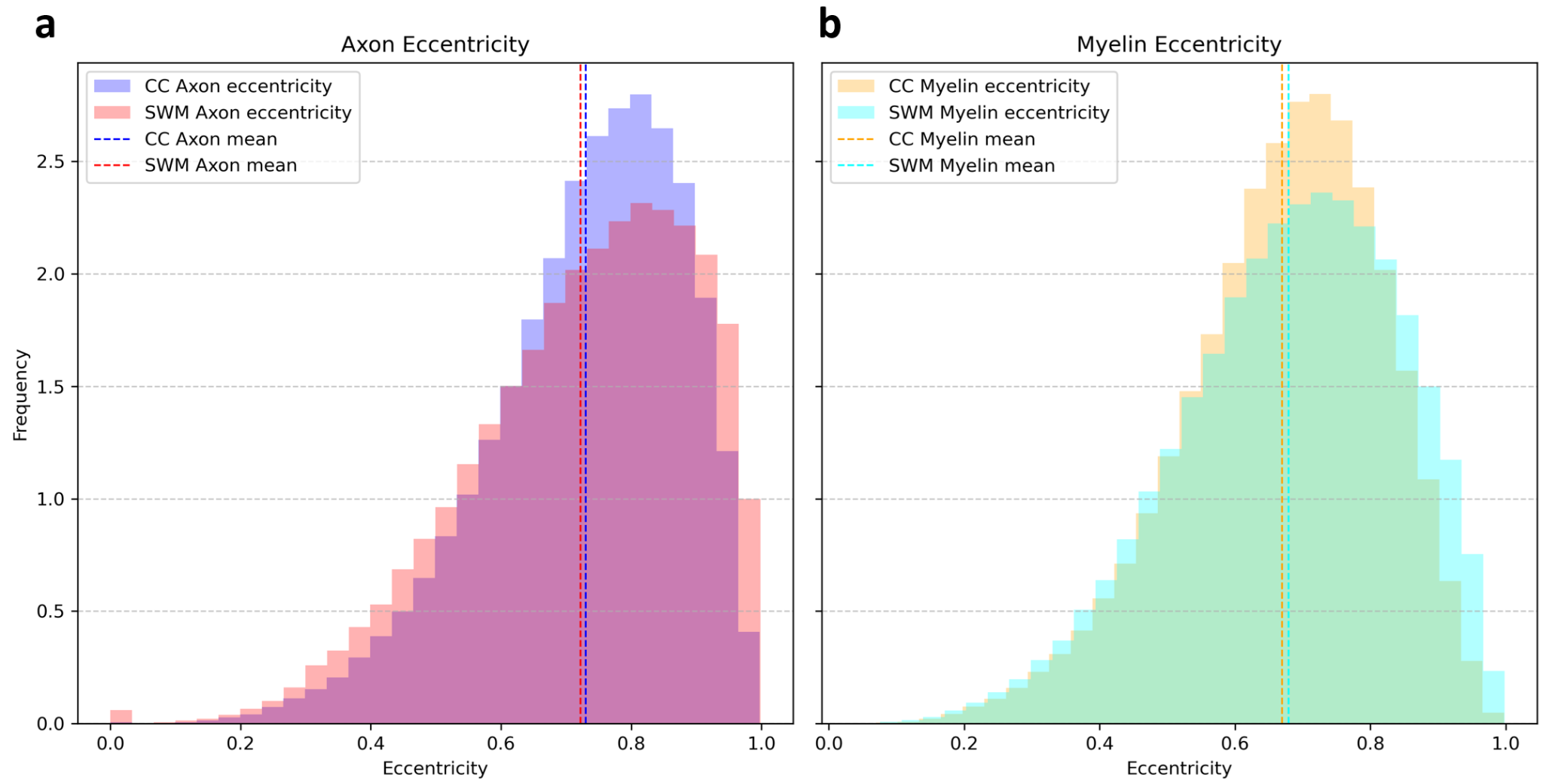

Supplement: S8 Fig — This plot shows the distribution of eccentricity of the ellipses that were fitted on structures (a: axons, b: myelin), and their means in four different subgroups: Both sampled regions (CC and SWM) and the measured structures. Means are 0.73 (CC axons), 0.72 (SWM Axons), 0.67 (CC Myelin), 0.68 (SWM Myelin). Data available in S3 Data (https://zenodo.org/records/15720452, [72]). (PNG) [file pbio.3002906.s008.png]

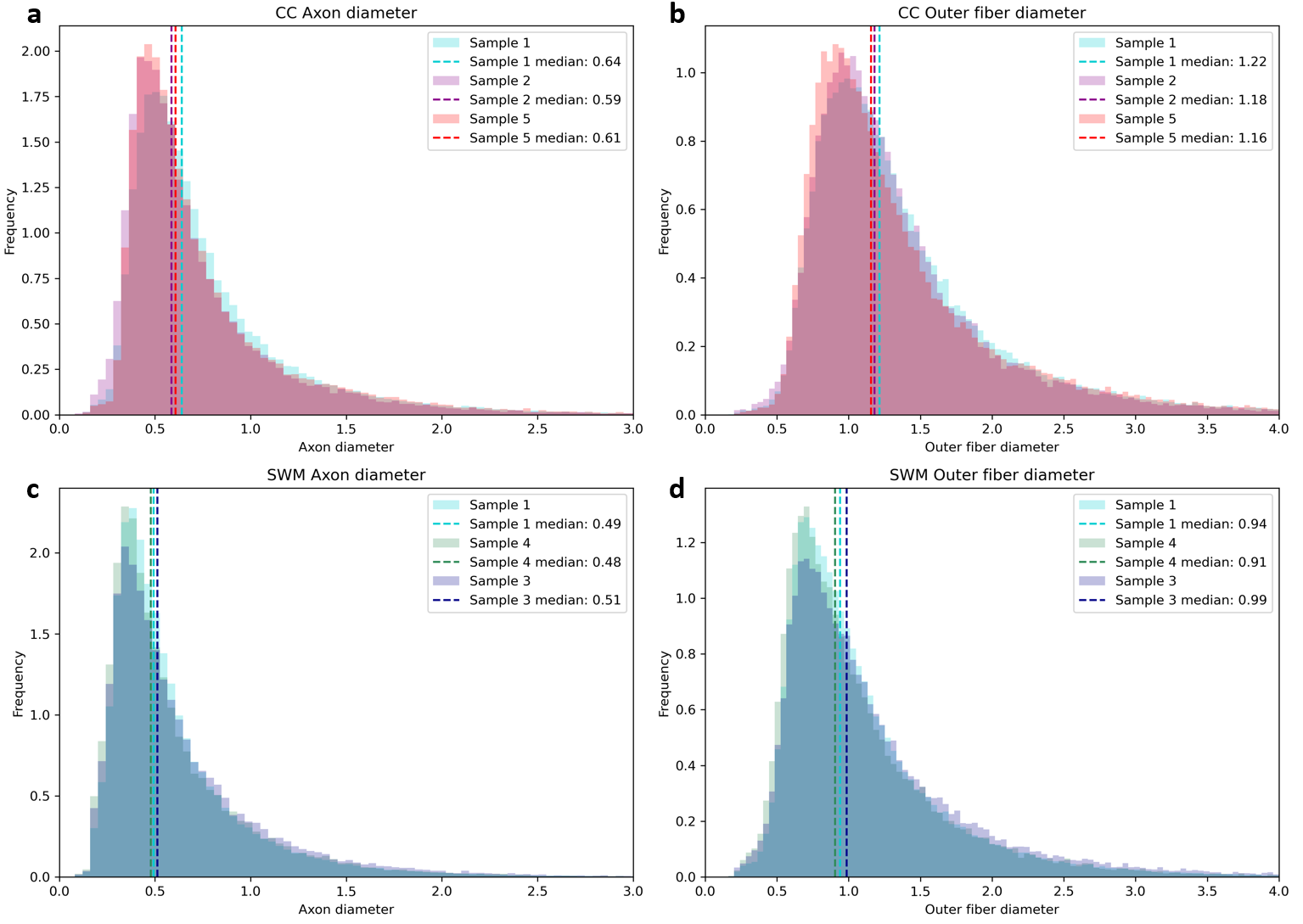

Supplement: S9 Fig — Each distribution shows the measured axon (a,c) or outer fiber diameter (b,d) in the CC (a,b) or SWM (c,d), respectively. Data available in S3 Data (https://zenodo.org/records/15720452, [72]). (PNG) [file pbio.3002906.s009.png]

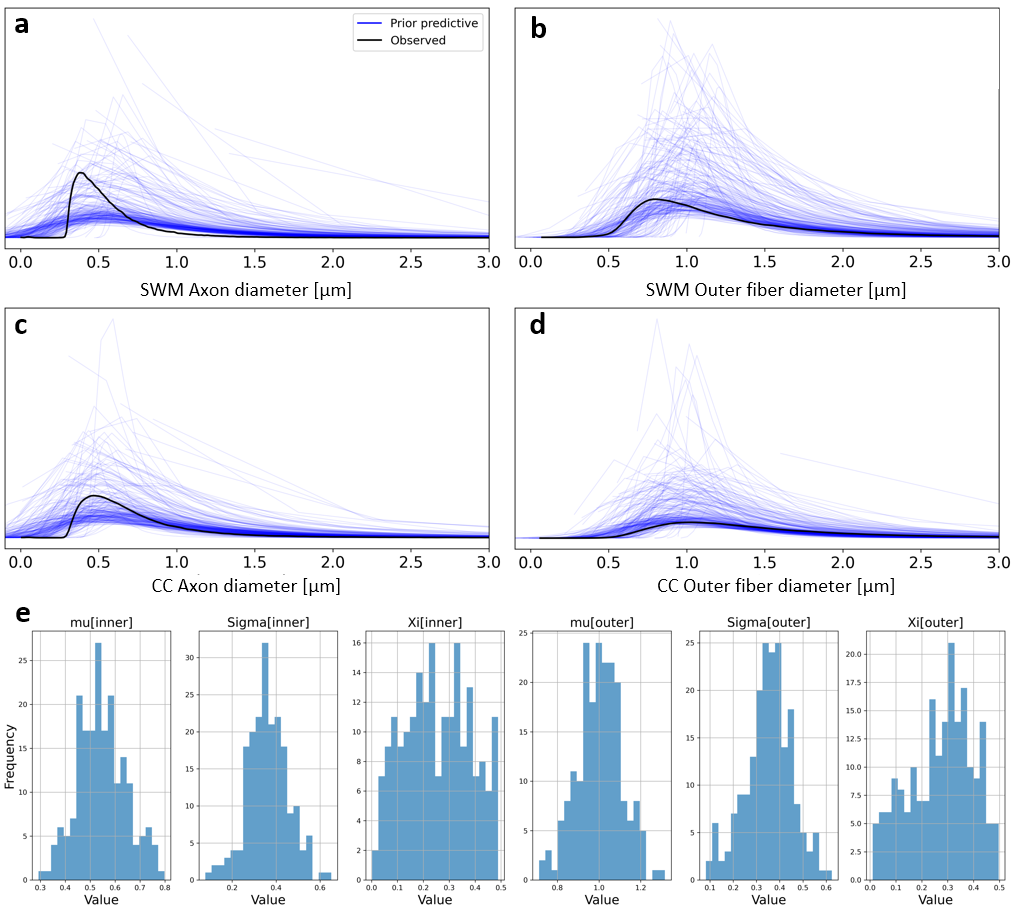

Supplement: S10 Fig — (a–d) show prior predictive GEVs for SWM axon diameter (a), SWM outer fiber diameter (b), CC axon diameter (c), and CC outer fiber diameter (d). (e) Histograms of 200 Prior samples for each of the parameters. Data is generated from prior assumptions. (PNG) [file pbio.3002906.s010.png]

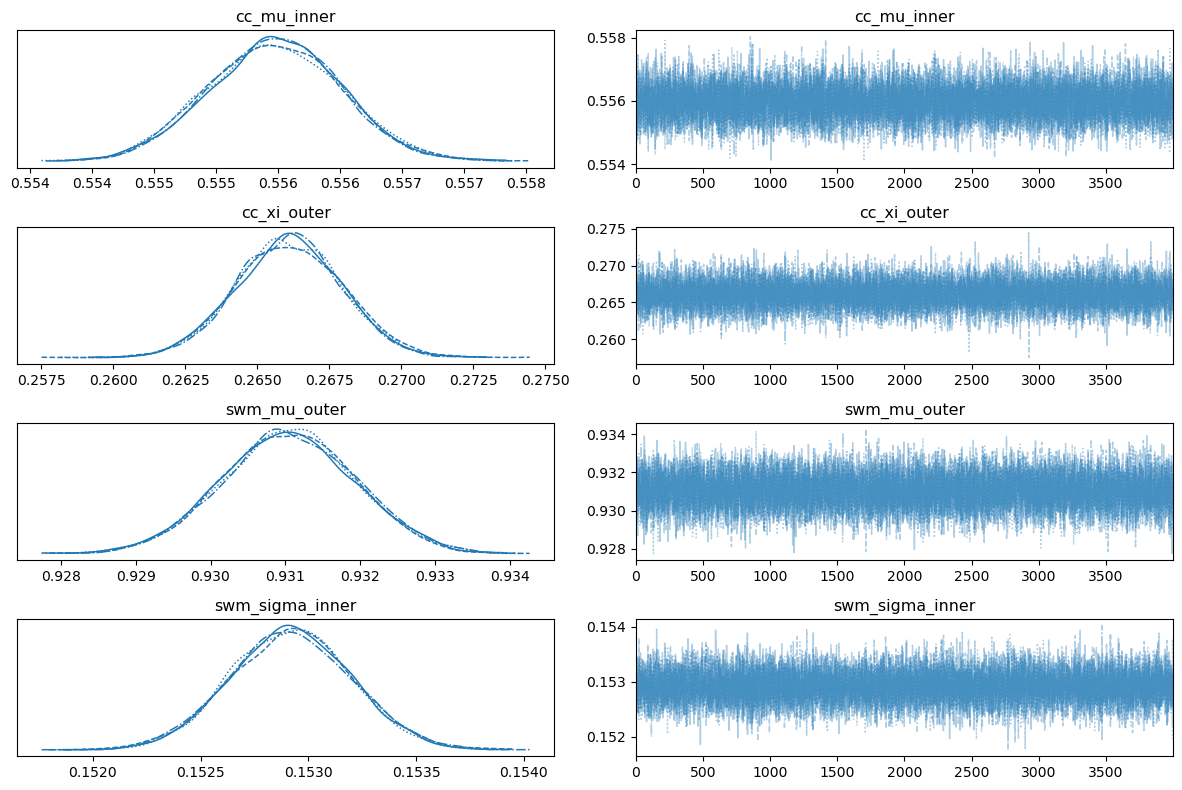

Supplement: S11 Fig — Left column shows histograms of all posterior samples for a set of four parameters. Different line styles represent 4 different chains. Right column shows trace plots of the MCMC sampling procedure for a single chain each. Data is not directly available due to data size limitations, but can be generated from running the MCMC script included in the github repository (mcmc_CCvsSWM_GEV.ipynb). (PNG) [file pbio.3002906.s011.png]

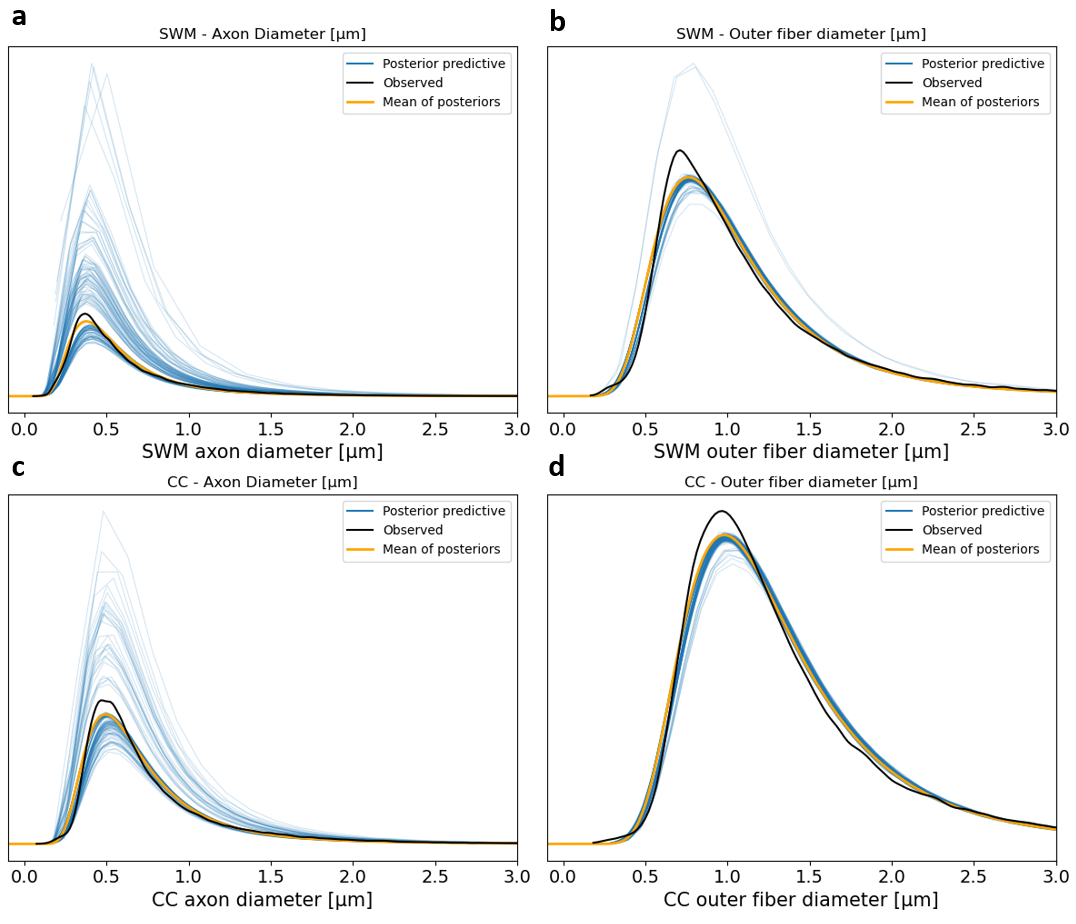

Supplement: S12 Fig — For each group of structures (CC and SWM, axon and outer fiber diameter), 150 randomly drawn posterior samples are drawn and plotted here (blue), alongside their mean (yellow) and the observed data (black). (a) shows CC axon diameter, (b) shows CC outer fiber diameter, (c) shows SWM axon diameter, (d) shows SWM outer fiber diameter. Data is not directly available due to data size limitations, but can be generated from running the MCMC script included in the github repository (mcmc_CCvsSWM_GEV.ipynb). (PNG) [file pbio.3002906.s012.png]
