# Supplementary material for: Short-range human cortico-cortical white matter fibers have thinner axons and are less myelinated compared to long-range fibers despite a similar g-ratio
Source: PLoS Biol. 2025 Aug 20;23(8):e3002906. doi: 10.1371/journal.pbio.3002906 (PMC12410883; doi:10.1371/journal.pbio.3002906)
Supplement: Attachment — Submitted filename: Ruthig_Edler et al_ Point by point response reviewers new.docx [file pbio.3002906.s016.docx]

Authors response to reviewers:

**We are grateful for considering our manuscript „Human short association fibers are thinner and less myelinated than long fibers” for resubmission to PLOS Biology. We are thankful to all reviewers who provided highly valuable comments, which we addressed point by point below.**

**In addition to the improvements suggested by the reviewers, we have revised the title as follows based on editorial recommendations: “Short-range human cortico-cortical white matter fibers have reduced axon diameter and myelination compared to long-range fibers, suggesting a fine tuning of conduction velocities. Furthermore, we have restructured the discussion. We now have introduced subheadings in the discussion to enhance the logical flow and minimize any potential lack of connectivity between sections.**

Reviewer #1: The authors present a study on the electron microscopic evaluation of differences in fiber diameter and myelin sheath thickness in callosal and short white matter fibers. The found thicker and more highly myelinated long range as compared to the short-range fibers, based on an automated deep learning-based segmentation of the electron microscopy data from human brain tissue samples in different subcortical white matter regions underneath primary motor and sensory regions and corpus callosum from 5 postmortem human brains.

The study addresses a very relevant and timely topic which provides a profound basis for connectomic analyses and understanding of differences in information transfer across brain regions.

I nevertheless have several things which shall be addressed:

*	Methods: The authors mention that they labelled 'about 16,000 cells' manually. Could the authors expand a bit more on what is meant by that? As the authors obviously did not label the cell bodies, but the axons, how could they assure that they labelled 16,000 different cells? And as this cannot mean that they labelled the whole axon of each cell, how did they assure that they did not label the same axon, and thus, same cell in consecutive sections?

**Response: *Calling myelinated fibers (consisting of a neuron + oligodendrocyte) ‘cells’ was an oversight. Therefore, we rephrased the respective text passage accordingly and now, more precisely, state that we labeled ca. 16000 two-dimensional sections of myelinated fibers.***

**Line 410: *We labelled ~16000 two-dimensional sections of myelinated fibers [...]***

***Due to the inherently two-dimensional nature of the method, we cannot entirely rule out the possibility that the same fiber appears multiple times in the data set. However, to mitigate this, we did not image consecutive sections of the same region. Instead, we used large two-dimensional fields of views (ca. 80 µm x 800 µm for each acquisition) and sampled tissue for each acquisition from locations spaced several millimeters apart. Consequently, we consider it unlikely that the same set of fibers are represented more than once in the dataset. However, in order to clarify this point, we now included this information in our methods section.***

**Line 386f.: [...] *we used large-two dimensional fields of views (ca 80 µm x800 µm) and sampled tissue for each acquisition from locations spaced several millimeters apart in order to decrease the likelihood of including individual fibers multiple times within the data set.***

*	Methods: The authors state in their section about validation that "we trained a validation model on a subset of randomly chosen labeled training images and validated its prediction against the last image not inside the subset." - what exactly is meant here with 'the last image not inside the subset' if the subset was chosen randomly from the whole number of images?

**Response**: ***In total, we had a set of 40 labeled images in our training dataset. We chose a subset of these images at random (for CC and SWM each) to leave out for the training of a separate validation model. Then, we validated the predictions of the validation model against the manually generated labels of these remaining images (which were not part of the training data for the validation model). The aim was to validate our model’s performance against the gold standard, which is expert annotation. We agree that the phrasing is unnecessarily complicated and simplified it in the text.***

**Line 440ff.: *For the validation of our segmentation pipeline, we trained a separate validation model. For the training of this validation model, we excluded a set of randomly chosen images from the training data (both from CC and SWM), and validated the predictions of the validation model against the manually generated labels for these excluded images.***

*	The authors mention several validation analyses for excluding biasing effects in the analysis and data in the main text, referring to the supplemental figures. It would be helpful to the reader if these additional analyses are explained somewhere similar to a 'main' analysis as the figures are hardly understandable as they stand now.

**Response: *We expanded the figure captions of the supplementary figures significantly, and also added a section about the included control analyses in the methods section, in order to better explain the validation procedures.***

**Line 439ff.: *chapter ‘Transmission Electron Microscopy analysis - Validation’***

*	Results / Discussion: The authors explain differences in their results in comparison to a previous study as potentially resulting from differences in measuring axon diameter (ellipse vs. circle). While this is certainly a valid potential explanation, one might wonder if the circle of the ellipse is the more reliable way of estimating a fiber's diameter, given the distortions in postmortem tissue vs. the real-life situation. To allow for direct comparison and estimation of the delta between these approaches, it might be worthwhile if the authors provide both measures.

**Response**: ***We thank the reviewer for this important comment and added a respective paragraph addressing this issue within our discussion:***

**Line 253ff.: *A matter of discussion in studies regarding myelinated fibers is the correct way of using a two-dimensional image to determine the diameter of a three-dimensional tubular structure, which is likely not perfectly orthogonal to the orientation of a two-dimensional image. In our case, we decided to fit ellipses on each structure, analogous to the morphometric measurements employed e.g. by Abdollahzadeh et al., (2019). We believe that this approach has two major advantages over alternative strategies: i) When cutting a tubular structure at an angle, an ellipse is created in the cutting plane. The minor axis of this ellipse (= the shortest distance between two sides of the ellipse passing through the center) is always equal to the true diameter of the three-dimensional tubular structure. This is especially useful in our case, as we expect a large amount of non-orthogonally cut fibers in the SWM. ii) Additionally, when structures are damaged (e.g. parts of myelin sheath missing or partly broken down), fitting an ellipse allows a reasonable estimate of the structure before degradation. A downside of this approach is that we cannot differentiate between a) round, but oblique cut fibers (which this method would accurately measure) and b) oval, but orthogonally cut fibers (where this method would underestimate the diameter of a structure).***

***We also implemented an alternative measurement method according to Behanova et al (2022), which relies on the equivalent diameter of a circle, dependent on the axonal cross section area. As expected, due to oblique/diagonal cuts increasing the cross-section area, we see a higher average axonal diameter when measuring according to the area-equivalent circle radius (Fig. S6) in CC (+27%) and SWM (+48%).***

*	Results: As one of the major results of the study, the authors emphasize the analysis on conduction velocity for which they now demonstrated differences between different brain regions with different types of fibers. While this is generally true, it needs to be mentioned that this result is mainly derived from a calculation based on an existing formula. There is no direct prove of differences in conduction velocity, though. Thus, I would not necessarily call this a direct result of the present study, but more of a possibility that could now be implemented in studies on conduction velocity.

**Response**: ***We thank the reviewer for this point and now more clearly state that the conduction velocities in Fig 3f are estimated (and not measured) within the figure, figure caption, and the methods section. In the discussion, we acknowledge that other factors than axon diameter and myelin thickness contribute to conduction velocity. More broadly, our aim was to present this finding precisely as the reviewer describes it: as an example of how other studies might potentially build upon or reuse our results. To align our motivation and the perception by readers, we rephrased sections of the text, including the heading of the respective result chapter.***

***Furthermore, we performed a plausibility check using the H01 dataset released by the Lichtman Lab (Shapson-Coe et al., 2024) by identifying two low-caliber SWM axons in order to compare the range of our g-ratio-based velocity estimates with estimates based on internode and node length from the Lichtman dataset.***

**Line 149: *Region-specific anatomical specialization might underlie conduction velocity changes***

**Line 154: *[…] we estimated conduction velocities according to Rushton’s formula […]***

*	Discussion: As one of the limitations, the authors stress the issue with working with human tissue and with artefacts arising from the processing of this tissue. As they used tissue from five body donors, it would be really helpful to see some comparative examples of tissue snippets and how they were processed with the automatic segmentation algorithm to have a better idea of the variability within the data and about the methodical variability.

**Response**: ***In fact, we did not observe a large inter-specific variability in our data, neither on a visual level nor in the measured data. The described problems seem to be universal for aged human tissue, given the used methods. To show this, we now include a figure (Fig. S9) showing the variability of the main measurements (axon + outer fiber diameter) in all different samples, showing very little variability between individuals. In all cases, the variability is substantially smaller than the described difference between CC and SWM fibers.***

Reviewer #2: In this paper, the authors assess the axon diameter and the myelination of long fibers in the corpus callosum and shorter fibers in superficial white matter. The axon diameter and myelin thickness of short fibers in human brains is currently not well described and this understanding is of high value to the field. The study assesses the axon diameter and myelin thickness in long fibers in the corpus callosum, and short fibers between sensory and motor areas and between V1 and V2. They used transmission electron microscopy in 5 older post-mortem brains and trained a U-net to evaluate 400.00 fibers. They find that the longer fibers are thicker and the shorter fibers are thinner, as is expected. They also assess the g-ratio and find that the g-ratio is comparable across the two fiber types with a value of ~0.6. This has important implications for understanding brain structure and function given the variable conduction velocities that these different fiber types can have.

Major:

As there could be potential bias of the U-net towards larger fibers, the performance of the U-net on the smaller fibers should be shown in the same was as in the example in Figure 2 (which is from corpus callosum data).

**Response**: ***We thank the reviewer for this point and comprehensively evaluated the performance of the U-net (in the revised manuscript, we now reference it as DenseNet) in our validation experiments on both CC and SWM fibers (Fig. S1-S5), including a measure that specifically measures inaccuracy depending on fiber size (Fig. S3,S4, Bland-Altman plots). This analysis revealed a good agreement between manually labeled data and the automated prediction, irrespective of the axon or outer fiber diameters.***

***In addition, we now expanded Figure 2, showing the same steps in SWM to give an intuitive impression of the performance of our analysis as well.***

How is the diameter calculated for fibers that have an ellipsoid shape, like many examples in Figure 2? What are the potential explanations for the ellipsoid shape?

**Response**: ***We likely do not orthogonally cut most fibers in our 2D data. In order to still reliably get the most accurate diameter of tubular structures from a 2D image, we fit an ellipse to each segmented structure and measure the shortest diameter of the ellipse - which is identical to the actual diameter of a tubular structure that is cut in a non-orthogonal 2D plane. We clarified this further in our methods section and in an additional discussion chapter on the topic (see also answer to the next question).***

The control for the parallel cuts is unclear and there seems to be no control for diagonal cuts. For the parallel cuts, figure S6 should show these data, and states that: "Two example slices for the parallel cut can be seen in Fig. S1 c,d. Orthogonal cuts are not shown." However, no such slices are shown. For diagonal cuts, it seems like a relatively diagonal slice could increase the estimated diameter while having a relatively minor effect on myelin thickness, but this does not seem to be the case for Figure S6. Is it possible to explain this? If a diagonal cut consistently leads to increased diameter estimates, this could systematically bias the results.

**Response**: ***We apologize that our phrasing of ‘parallel’ and ‘orthogonal’ cuts has been confusing in the original version of the manuscript. To clarify: In our parallel cuts, we aimed to cut in parallel to the main fiber direction, generating longitudinally/diagonally cut fibers. In another subset of samples of the same tissue, we cut orthogonally to the main fiber direction. To control if the cutting angle is a factor for the measured axon/myelin diameters, we show the resulting distributions of parallel/orthogonal cuts in Fig. S7 (previously Fig. S6). To make the concept more intuitive, we added new subpanels to Fig. S7, showing the respective cutting angles and the main fiber direction in a histological slice. Furthermore, we expanded the figure caption of Fig. S6 and our methods section, explaining the approach. Additionally, we rephrased the main text sections to be clearer, stating that parallel and orthogonal is in respect to the main fiber direction in SWM.***

**Line 338ff.:** ***A subset of the data from SWM was cut orthogonal to the main fiber direction (U-shape perpendicular to the sulcus), and another subset was cut in parallel to the main fiber direction to check for a potential bias with respect to the cutting angle (Fig. S7).***

**Line 253ff.:** ***A matter of discussion in studies regarding myelinated fibers is the correct way of using a two-dimensional image to determine the diameter of a three-dimensional tubular structure, which is likely not perfectly orthogonal to the orientation of a two-dimensional image. In our case, we decided to fit ellipses on each structure, analogous to the morphometric measurements employed e.g. by Abdollahzadeh et al., (2019). We believe that this approach has two major advantages over alternative strategies: i) When cutting a tubular structure at an angle, an ellipse is created in the cutting plane. The minor axis of this ellipse (= the shortest distance between two sides of the ellipse passing through the center) is always equal to the true diameter of the three-dimensional tubular structure. This is especially useful in our case, as we expect a large amount of non-orthogonally cut fibers in the SWM. ii) Additionally, when structures are damaged (e.g. parts of myelin sheath missing or partly broken down), fitting an ellipse allows a reasonable estimate of the structure before degradation. A downside of this approach is that we cannot differentiate between a) round, but oblique cut fibers (which this method would accurately measure) and b) oval, but orthogonally cut fibers (where this method would underestimate the diameter of a structure).***

***We also implemented an alternative measurement method according to Behanova et al (2022), which relies on the equivalent diameter of a circle, dependent on the axonal cross section area. As expected, due to oblique/diagonal cuts increasing the cross-section area, we see a higher average axonal diameter when measuring according to the area-equivalent circle radius (Fig. S6) in CC (+27%) and SWM (+48%).***

**Line 463ff.: *We cut approximately half of our SWM samples in parallel to the main fiber direction (U-shape perpendicular to the sulcus), as described by blunt dissections*** [***(e.g. Catani et al., 2012)***](https://www.zotero.org/google-docs/?QLFW77)***, and the other half orthogonally to the main fiber direction. We show the resulting axon and outer fiber diameters and corresponding histological sections in Fig. S7.***

Minor:

It would be helpful if the abstract stated the method used for the measurements.

**Response**: ***We thank the reviewer for this point and added the information to the abstract (line 29f.).***

Figure 1C is missing a scale bar.

**Response**: ***The scale bar we had in Figure 1f applied to 1c as well, but we did not state that clearly. We now added an identical scale bar to 1c.***

The variability between subjects should be shown.

**Response: *We thank the reviewer for this important point. We now added a figure showing variability between subjects (Fig. S9). The plot demonstrates that the variability between subjects is substantially smaller than the described difference between SWM and CC.***

Was there and variability between V1-V2 and sensory-motor fibers?

**Response**: ***The present article is designed to be a discovery report, and is focused on the central finding in our data, which is the difference between two major groups of white matter fibers, CC and SWM. We plan to explore more local differences within SWM in a follow-up article, including differences between V1-V2 and sensory-motor fibers. The investigation of this question is ongoing and will likely be part of a respective update article.***

Reviewer #3 (Jussi Tohka): The manuscript describes a computational pipeline and experiments on transmission electron microscopy (TEM) images of the human brain. It applies a standard deep learning-based segmentation technique (U-Net semantic segmentation followed by watershed for instance segmentation) to segment axons and their myelin sheaths from TEM images of five donors. The manuscript concludes that there are substantially smaller fiber diameters and lower myelination in superficial white matter compared to the corpus callosum, while the g-ratios are nearly equal. I was tasked with evaluating the technical aspects of the manuscript.

The segmentation of 400,000 axons in human-derived data represents a comprehensive piece of work that has the potential to generate new insights into the organization of the human brain. I have a few technical questions, concerns, and discussion points, as well as some editorial suggestions (point 6). I think that the proper answer to question 4 could provide a proxy to "independent validation". Answers to each of the comments 1 - 5 are essential to ensure the technical validity and rigor of the work

1. If I understood correctly, all the measurements were performed on 2D slices. How does this influence the results, especially considering that axons do not all align according to the imaging plane? Some discussion on this matter would benefit the manuscript. Figure S6 provides some insights, but I do not understand why orthogonal cuts are not shown.

***Response: We thank the reviewer for this point. Indeed, all data used in this study is two-dimensional and we now added a respective paragraph in the methods section to clarify this point. We also added histological slices of orthogonal and parallel cuts to Fig. S7 (Fig S6 in the initial submission), showing the respective cutting angles we used to control for this potential bias. As suggested by the reviewer, we now also added a paragraph to the discussion section that addresses how the two-dimensional nature of our data may influence the obtained results. This discussion paragraph reads as following:***

**Line 253ff.: *A matter of discussion in studies regarding myelinated fibers is the correct way of using a two-dimensional image to determine the diameter of a three-dimensional tubular structure, which is likely not perfectly orthogonal to the orientation of a two-dimensional image. In our case, we decided to fit ellipses on each structure, analogous to the morphometric measurements employed e.g. by Abdollahzadeh et al., (2019). We believe that this approach has two major advantages over alternative strategies: i) When cutting a tubular structure at an angle, an ellipse is created in the cutting plane. The minor axis of this ellipse (= the shortest distance between two sides of the ellipse passing through the center) is always equal to the true diameter of the three-dimensional tubular structure. This is especially useful in our case, as we expect a large amount of non-orthogonally cut fibers in the SWM. ii) Additionally, when structures are damaged (e.g. parts of myelin sheath missing or partly broken down), fitting an ellipse allows a reasonable estimate of the structure before degradation. A downside of this approach is that we cannot differentiate between a) round, but oblique cut fibers (which this method would accurately measure) and b) oval, but orthogonally cut fibers (where this method would underestimate the diameter of a structure).***

***We also implemented an alternative measurement method according to Behanova et al (2022), which relies on the equivalent diameter of a circle, dependent on the axonal cross section area. As expected, due to oblique/diagonal cuts increasing the cross-section area, we see a higher average axonal diameter when measuring according to the area-equivalent circle radius (Fig. S6) in CC (+27%) and SWM (+48%).***

2. The computation of the g-ratio by fitting ellipses (likely not ellipsoids) seems overly simplistic given the non-ellipse-like cross-sections of the axons. The mean myelin thickness can be easily obtained by a distance transform and compared to the square root of the cross-sectional area of the axons, as done elsewhere. Would this change the results? Specifically, are the cross-section shapes similar between superficial white matter (SWM) and the corpus callosum (CC)?

**Response**: We thank the reviewer for this point, and for highlighting the ***typo (ellipsoid/ellipse) in the Figure 2 caption. Our main reason for fitting ellipses to each structure is that when we fit an ellipse to a 2D section of a tubular structure and measure the shortest diameter of the ellipse, the shortest diameter of the ellipse is identical to the actual diameter of a 2D section of a tubular structure, irrespective of the cutting angle (see also comment above). We expanded our methods and discussion sections to include these points and make our approach clearer. Furthermore, the myelin sheath is frequently not intact, given the sample conditions (aged human post mortem tissue). When fitting an ellipse, the missing parts of myelin are relatively robustly estimated, whereas the distance transform of a structure with irregular boundaries might be more severely impacted by these artifacts. While the proposed solution is feasible for e.g. mouse data of higher quality and precise orthogonally cut nerve strands (or 3D data, that can be oriented so the virtual cutting plane is directly orthogonal to the structure), it is in our opinion not the method of choice for our data. Nevertheless, we replicated the morphology measurement strategies used e.g. in Behanova et al (2022) and compared them to our own in Fig. S6. As expected, measuring the axonal diameter via the diameter of the area-equivalent circle enlarges the measured axon diameters due to oblique cuts (Fig. S6 a,c). Due to the higher amount of oblique cut fibers in SWM, this region is more strongly affected: while the CC exhibits a +27% increase in axon diameters when measured using an area-equivalent circular approximation, SWM shows a mean increase of +48%. The proposed combination of maximum distance transforms and skeletonization modifies the measured outer fiber diameters only marginally. Although we consider both methods viable and robust, the correction of oblique cuts leads us to prefer the more straightforward approach of ellipse fitting in this case.***

***We assume the second point the reviewer makes concerns the eccentricity of fitted ellipses. We already include an overview of how eccentricity varies (mean+-sd) in the different groups (axon/myelin in CC/SWM) in Figure S1 and S2. To be more comprehensive in this point, we added another supplementary figure (Fig. S8) showing the full eccentricity distributions of these four groups.***

**Line 426ff.**: ***Due to the sample conditions (aged human post mortem brain tissue), fibers are frequently damaged. Therefore, we decided to not directly measure the segmented binary structures, but measure fitted ellipses to each structure with the same normalized second moment. This solution ensures that even damaged structures (e.g. sections of myelin sheath missing) are estimated reasonably well. In addition, this solves the problem of oblique cuts: The minor axis of an elliptical cut of a tubular structure is identical to the true diameter of a tubular structure, irrespective of the cutting angle.***

3. If I understood correctly, the evaluations in the supplementary figures were done using a leave-one-donor-out approach. Please confirm. It would be instructive to see image segmentation performance measures in addition to the Intersection over Union (IoU). I would prefer to see the average Hausdorff distance, as it is easier to interpret in this context.

**Response**: ***In total, we had a set of 40 labeled images in our training dataset. We chose a subset of these images at random (for CC and SWM each) to leave out for the training of a separate validation model. After, we validated the predictions of the validation model against the manually generated labels of these remaining images (which were not part of the training data for the validation model). We added the Hausdorff distance to our validation metrics and now include the 95th percentile Hausdorff distance in our validation data (ca 150x150 µm: SWM-axons: 0.37 µm, SWM-myelin: 0.41 µm, CC-axons: 0.04 µm, CC-myelin: 0.06 µm. Furthermore, we also added the Dice/F1 score to the manuscript (0.94/0.91 for CC axons/myelin, 0.77/0.70 for SWM axons/myelin).***

4. The statistical analysis is the most concerning part of the manuscript. It appears that the manuscript aggregates the measurements from five donors without analyzing whether this is appropriate or if a hierarchical modeling approach should be used instead. There are many possibilities to demonstrate this and perform the hierarchical analysis if needed, so I leave it to the authors to decide on the best approach. Additionally, the manuscript's statements about the results are descriptive. While I do not think p-values or similar metrics are necessary, the manuscript should quantify the sizes of the differences in measures between the CC and SWM. It might also be beneficial to soften the title of the manuscript.

**Response**: ***We thank the reviewer for the valuable technical feedback. We also thank the reviewer for not forcing p-values into a manuscript that does not require them. We now include a figure displaying the donor-split measured data (Fig. S9). While the concern for a high deviation between donors is certainly valid, in this case the distributions overlap in a major way and their variation is significantly lower than the difference we quantified between the different regions. Additionally, since we are employing a Bayesian approach, a wide split of the raw data over different samples would also be reflected in uncertainty in our posterior distributions, which are however fairly sharp (see 99% HPD intervals in Fig. 4b). Therefore, we chose not to apply a hierarchical model in this context. To assess model fit, we conducted posterior predictive sampling (Fig. S12), which shows that the posterior means closely capture the characteristics of the observed data.***

5. I would welcome more detail about the watershed method used after the semantic segmentation. It is unclear whether the manuscript trains a U-Net, DenseNet, or a combination thereof. Please clarify. Additionally, information about the network training, including its specific architecture, loss function, most important hyperparameters, and optimization algorithm, should be provided (this can be included in the supplement). Why was the data downsampled? What threshold was applied?

**Response**: ***We used a singular DenseNet, and refrain from calling it a U-Net in the text now to avoid potential confusion. We added all parameters, the used loss function, and network architecture variables used to configure the training in Uni-EM to our methods section. We expanded the description of the used watershed method in our methods section, see below. We downsampled the data to reduce the training and inference times of the algorithm - if we hadn’t done that, training would have taken months instead of weeks on a desktop GPU workstation (NVIDIA QUADRO RTX 5000). Since the structures we aimed to segment with this network are rather big for the method (smallest fibers are ~800x800 pixels in the original data), performing the analysis on a factor 4 x and y downsampling is sufficient for this task. The applied thresholds to create binary images from the prediction image were manually selected. Furthermore, we now go into more detail in the text about the watershed method employed.***

**Line 419ff.:** [...] ***These pictures were then predicted by the trained DenseNet to yield a semantic segmentation (Fig. 2b, e). This semantic segmentation is thresholded to generate binary masks for a) axons and b) myelin. In order to separate ‘kissing’ myelin sheaths, these masks are joined together and a distance transformation is performed. The inverse of this distance transform is used to perform a watershed method, with seeds at every peak of the distance transform, generating a unique identifier for each axon+myelin pair. After labeling every fiber with a unique identifier, the fiber is split back to its axon and myelin portions using the binary masks generated before (Fig. 2c, f).***

6. The manuscript suffers from some sloppy writing. For example, phrases like "White matter connects neighboring and distant cortical areas," "pixel resolution," "human samples," and "by matching instances with >40% identity" require rephrasing. I would prefer the results to be presented in the past tense, as they relate to these particular experiments, not general facts. Also, I found references to "a fundamental principle" unclear because it is not specified what principle is being referred to. I think that the distribution function of GEV has a typo in it.

**Response**: ***We changed the given expressions accordingly, and changed the results section to be in the past tense, where applicable. Thank you for finding the typo in the GEV function. We also rephrased the explanation for the fundamental principle we are describing: Axon diameters and myelination thickness in white matter fibers.***

**Line 40: *Myelinated fibers in the white matter connect both nearby and distant cortical areas.***

**Line 67ff.: *In this study, we investigate this fundamental principle of white matter fiber organization. Specifically, we analyze the axon diameter and myelination thickness in regions with either predominantly short or predominantly long-range fiber connections.***

**Line 84ff.: *In the present study, we employ a state-of-the-art automated segmentation algorithm to analyze large quantities of high-quality human tissue samples. In this study, we show that short association fibers have smaller axon and outer fiber diameters compared to long-range fibers. We provide experimental evidence for the distinct structural characteristics of short association fibers in the human brain and highlight the tight link between function and structure in different white matter fiber tracts.***

**Line 450ff.: *To check if specific fibers are distorted in a certain way in the prediction compared to the manually labeled data, we matched each predicted fiber with its corresponding manually labeled fiber in the validation data (fibers with >40% overlapping areas are treated as the same fiber). All fibers that had no corresponding fiber (<40% overlap) in the prediction or validation data were treated as false positives or negatives, respectively.***

*We thank Jussi Tohka and both anonymous reviewers for the extensive and detailed technical and scientific feedback - it certainly helped substantially in improving the quality of our manuscript.*
